# Cooking Economies

**Published:** 1871-08

**Authors:** 


### COOKING ECONOMIES.

THE French are the most economical people in the world in the kitchen; more than any other nation they make the most out of little; no scrap is lost, and delicious dishes are made of what many would not think worth while to pick up from the table after the meal is over.

Americans use immense amounts of butter in the kitchen, costing all the way up to seventy-five cents a pound; butter and lard seldom go into a Frenchman's kitchen. Every atom of the juices of all meats is carefully preserved, and is made to subserve the place of butter in almost all cooking processes.

A pound of meat in one piece will not make much soup in an hour's boiling; if cut up into many small pieces, it does better, and better still if boiled gently for hours; this is also the best method of getting the substance out of all vegetables. Hence a Frenchman's wife puts a pot of water on the fire every morning, and keeps it simmering all day; and all day long she puts into it every little bone, every piece of vegetable, every scrap of meat, that can be gathered from the plates after eating, and there it simmers and simmers hour after hour all day long; then, a little while before wanted for use, give the whole a short, brisk boil; throw in a little cold water now and then to make it clear, keep the lid on, to save all the essence which would otherwise escape in the form of the odor of the soup kettle, and with the other ordinary "fixings" the whole becomes delicious, and so substantially nutritive as to be very valuable for the table in several forms.

Hominy, made by cracking each grain of Indian corn into several pieces, is made perfectly delicious, if soaked in warm water all night, and simmered all the next day, with a good boil before using; it can be eaten to great advantage with salt or pepper or butter or syrup; a bowl of rich, fresh, sweet milk, with hominy to suit, prepared as above, will make a breakfast or an early supper, sufficient and good enough for anybody.

The waste and hurry of American cookery is amazing to foreigners. It ought to be a distinct branch of study for our daughters how to get the most nutriment out of every article of food, and to prepare it in such a manner as will give the stomach the least work in fitting it to feed the body.

